# *Pseudomonas aeruginosa* infection in respiratory samples in children with neurodisability—to treat or not to treat?

**DOI:** 10.1007/s00431-021-04025-y

**Published:** 2021-04-06

**Authors:** Elizabeth Gregson, Lowri Thomas, Heather E Elphick

**Affiliations:** Department of Respiratory Medicine, Sheffield Children’s NHS Foundation Trust, Sheffield, UK

**Keywords:** *Pseudomonas aeruginosa*, Child, Neurodisability, Antibiotic resistance, Point-of-care testing

## Abstract

The objective was to investigate the prevalence of *Pseudomonas aeruginosa* (PA) in patients with complex neurodisability and current treatment practice in our centre in order to inform future guidelines. A retrospective case note review was undertaken at a tertiary children’s hospital. One hundred sixty-two patients (mean age 11.7 years) with a primary diagnosis of neuromuscular disease (NMD) or severe cerebral palsy (CP) and a respiratory sample sent for analysis during the study period were studied. Associations between PA in respiratory samples and diagnosis, long-term ventilation, presence of a gastrostomy or a tracheostomy, antibiotic choice, clinical deterioration and adverse events were analysed. Twenty-five (15%) had one or more PA isolate in respiratory samples. There was a significant association between PA in respiratory samples and tracheostomy (*p*<0.05). In 52% samples, multiple pathogens co-existed. There was no significant association between choice of antibiotic and clinical outcome but when antibiotics were changed to specific PA antibiotics during the course of the illness, all resulted in clinical improvement. Twenty-six episodes involving 8 patients with recurrent admissions involved PA organisms that were resistant to one or more antibiotics.

*Conclusions*: A larger prospective study may establish clearer criteria for guideline development. Techniques such as point-of-care testing to identify virulent strains of PA may improve patient outcomes and prevent the development of antibiotic resistance in the future.

**Table:** 

**What is Known:** *•Children with complex neurodisability are at increased risk of respiratory morbidity and of infection with gram-negative organisms such as Pseudomonas aeruginosa.* *•There are currently no guidelines to inform treatment choices in this group of vulnerable children.*	
**What is New:** *•15% children in this study population had Pseudomonas aeruginosa in respiratory samples during a 12-month period, the majority of whom did not require critical care treatment. Thirteen of these children had a tracheostomy in situ and 12 did not. * *•In those that deteriorated clinically or developed antibiotic resistant organisms, earlier detection and targeted treatment of Pseudomonas aeruginosa may have prevented deterioration.*

**What is Known:**

*•Children with complex neurodisability are at increased risk of respiratory morbidity and of infection with gram-negative organisms such as Pseudomonas aeruginosa.*

*•There are currently no guidelines to inform treatment choices in this group of vulnerable children.*

**What is New:**

*•15% children in this study population had Pseudomonas aeruginosa in respiratory samples during a 12-month period, the majority of whom did not require critical care treatment. Thirteen of these children had a tracheostomy in situ and 12 did not. *

*•In those that deteriorated clinically or developed antibiotic resistant organisms, earlier detection and targeted treatment of Pseudomonas aeruginosa may have prevented deterioration.*

## Introduction

Children with complex neurodisability such as neuromuscular disorders (NMD) and cerebral palsy (CP) are at increased risk of respiratory morbidity due to factors such as gastro-oesophageal reflux, kyphoscoliosis, muscle weakness, secretions and poor cough [1, 2]. 25% patients with cerebral palsy of severity 4–5 on the GMFCS (Gross Motor Function Classification System) have chronic respiratory problems [3] including cough and wheeze, obstructive sleep apnoea, cough on drinking and respiratory signs on examination [4]. Pneumonia, often due to aspiration, is a common cause of hospital admission, intensive care admission and death in these patients [5–8]. The more severely affected children are admitted 7 times more often, with 9.5 times as many admitted days as the normal population [9] Data suggest that pneumonia is responsible for 39% deaths in children with CP [10].

*Pseudomonas aeruginosa* (PA) is a gram-negative bacillus and is well known to cause infections and lead to colonisation of the airways. In Cystic Fibrosis, PA has been shown to bind to the respiratory epithelium [11–14]. It has been postulated from this evidence that PA also binds to the respiratory epithelium eroded by chronic subclinical oral aspiration and/or reflux causing chronic PA lower airway infection in patients with neurodisability [15]. There is a higher incidence of PA in children with CP in paediatric intensive care units (PICU) [10]; children with CP who are infected with PA are significantly more likely to have severe illness, be admitted to PICU and are also more likely to have prolonged or recurrent hospital admissions [15]. A review of children in PICU in Liverpool, UK reported that 89% children with CP carried PA or *Klebsiella*, compared with 55% without CP and 47% carried antibiotic resistant bacteria [10]. Treatment of PA in PICU is often problematic and the emergence of resistant organisms can occur as early as 8 days following admission [16].

Treatment for acute PA infection requires either a specific oral antibiotic, such as ciprofloxacin, which can exacerbate seizures or alter feeding regimes, or intravenous antibiotic treatment, which requires cannulation and hospitalisation. Both options lead to potential additional morbidity for the patient, including antimicrobial resistance [17]. Decision to treat with intravenous antibiotic treatment at an early stage in the illness may later prove unnecessary if the infection was in fact virus-driven. Conversely, delayed treatment for PA infection can result in severe clinical deterioration and escalation of treatment requirements. There are no universally agreed guidelines on when to treat PA, treatment choices are often empirical [18] or extrapolated from other conditions with a more established evidence base, for example cystic fibrosis [19]. There is a wide range of practice in terms of treating PA, with some clinicians treating only if symptomatic, some not treating at all and others treating regardless of symptoms [20].

The aim of this study was to investigate the prevalence of PA in patients with complex neurodisability and current treatment practice in our centre in order to inform future guidelines. Associations between clinical factors and treatment choices were analysed to establish whether these influenced clinical outcomes during an acute respiratory exacerbation.

## Methods

### Study population

The study population was all patients in the local region of our NHS Trust who:

1. Had a diagnosis of NMD or CP GMFCS 4 or 5
2. Did not have a primary diagnosis of cystic fibrosis or chronic lung disease
3. Had a microbiology culture on a respiratory sample during the study period

A comprehensive list of children with neuromuscular disease and cerebral palsy GMFCS 4 or 5 under the care of our NHS Trust was obtained from three databases kept up to date by the physiotherapy team working with children with neurodisability. A further database of children using overnight long-term ventilation (LTV) was obtained from the nursing team working with this patient group.

A list of patients that had a positive growth of PA on sputum or airway secretions between 01/01/17 and 31/12/17 was obtained from the microbiology lab records and amalgamated with the patient databases to find patients that fulfilled the study criteria.

### Data collection

The study was undertaken during the period 01/01/17–31/12/17 at a UK tertiary children’s hospital. All patients listed in the database who had also had a microbiology sample positive for PA during the study period were reviewed to elicit treatment decisions and clinical outcomes.

Data collection was as follows:

- Demographic information—age, gender, primary diagnosis, presence of tracheostomy, gastrostomy, use of LTV;
- PA infection—source of isolation; evidence of pre-existing infection/colonisation such as long-term antibiotics; other pathogens isolated during the illness being investigated;
- Treatments used, including type of antibiotic, timing relative to isolation of PA and antibiotic changes;
- Clinical information relating to a clinical deterioration or improvement, change in clinical condition after antibiotic changes;
- Adverse events as a result of treatment—side effects to antibiotics, organism resistance.

Some patients had several samples taken within the 12-month study period. A “PA positive clinical episode” was therefore defined as one or more positive isolates of PA within 1 week, as a surrogate measure of a single acute clinical illness. Samples that were positive more than 1 week apart were assumed to be a separate illness and/or a colonisation.

“Clinical deterioration” was defined as a persistent increase (for more than 12 h) of oxygen requirements, ventilatory support, any inotropic support/fluid resuscitation. If the PA was not treated and a clinical deterioration occurred within a week of this decision being made, this was counted as a deterioration due to no treatment being given. The term “treatment” is used specifically to only mean antibiotic treatment. Additional treatment for example supportive measures or respiratory support was not looked at specifically but only to establish if there was clinical deterioration. Treatment categories were antibiotics that are used specifically to treat PA such as ciprofloxacin or tazocin or non-specific antibiotics such as amoxicillin or azithromycin that may be used for a lower respiratory tract infection but not specifically to cover PA. This definition was chosen to apply standardisation within the cohort of patients analysed, given the retrospective design of the study.

### Data analysis

The anonymised data collection was stored in an Excel spreadsheet. Chi-square analysis was used to test whether diagnostic and clinical variables were statistically independent of one another (Excel). Analyses for type of antibiotic and clinical outcomes were repeated for patients without a tracheostomy. Statistical significance was considered to be at the level of *p* <0.05.

## Results

**Fig. 1:**
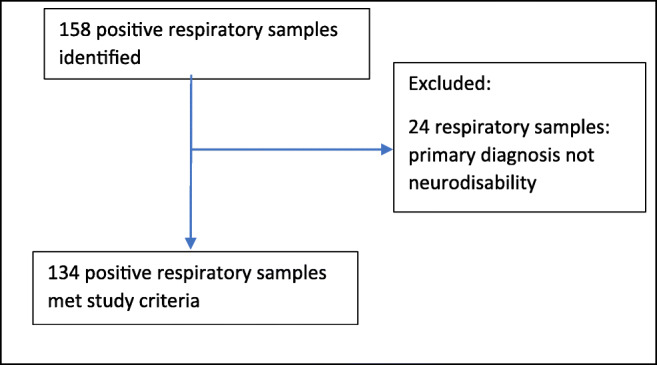
Flowchart of the positive samples identified in the microbiology lab records during the 12-month study period

Two hundred twenty-six patients were identified from the clinical databases and 158 positive samples for PA were identified in the microbiology lab records (Fig. 1). Patients with cystic fibrosis were excluded prior to searching the database; however, a further 24 patients were excluded as the primary underlying diagnosis was not a neurodisability. Therefore, 134 positive samples fulfilled the study criteria, with isolates grown from tracheostomy secretions, endotracheal tube secretions, nasopharyngeal aspirate or secretions, cough swab or sputum.

**Fig. 2:**
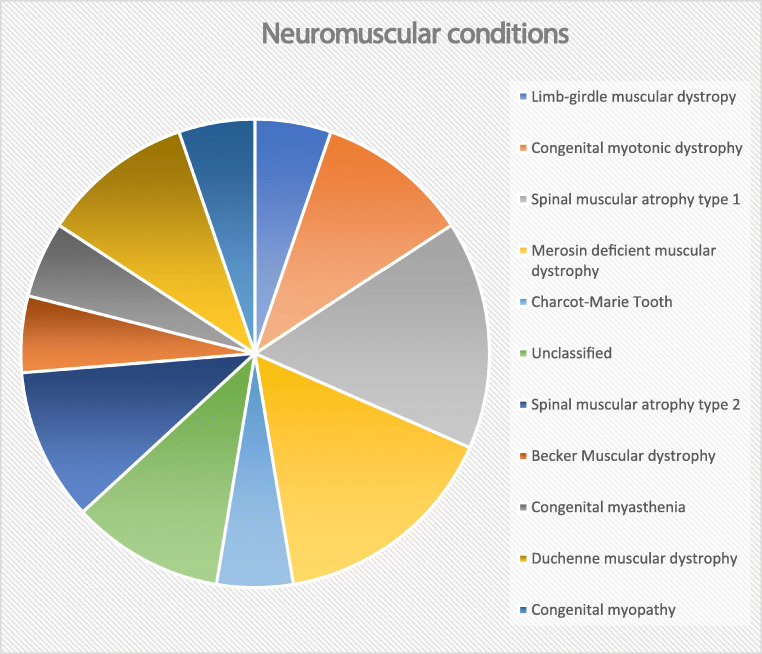
Neuromuscular diagnoses in patients infected with PA in respiratory samples (*n*=19)

Of the 181 patients with neuromuscular disease, respiratory samples were sent for bacterial analysis from 128 patients (71%). Nineteen of these patients had positive samples for PA (15%), of whom 11 (58%) were positive on more than one isolate within the 12-month period. Underlying conditions in the 19 patients with positive samples are shown in Fig. 2 and were muscular dystrophies: limb girdle muscular dystrophy (*n*=1), merosin-deficient muscular dystrophy (*n*=3), Duchenne muscular dystrophy (*n*=2), Becker muscular dystrophy (*n*=1); Spinal muscular atrophy type 1 (*n*=3), Spinal muscular atrophy type 2 (*n*=2), unclassified (*n*=2), congenital myasthenia (*n*=1), congenital myotonic dystrophy (*n*=2), Charcot-Marie Tooth (*n*=1) and congenital myopathy (*n*=1).

A total of 113 patients with cerebral palsy were identified. Of these, 45 had a GMFCS score of 4–5 and of these, respiratory samples were sent for bacterial analysis during the study period from 34 (76%). Six patients had positive samples for PA (18%), of whom two had more than one positive isolate within the 12-month period.

Table 1 shows the analysis of patients in whom a respiratory sample was taken during the study period (*n*=162).

**Table 1:** Analysis of patients in whom a respiratory sample was analysed during the study period (*n*=162). *NMD*, neuromuscular disease; *CP*, cerebral palsy; *GMFCS*, Gross Motor Function Classification System; *PA*, pseudomonas aeruginosa; *NIV*, non-invasive ventilation

Patient groups	PA positive	PA negative	*p* value (chi-square)
Total patients (*n*=162)	25/162 (15%)	137/162 (85%)	
Mean age (years)	13.7	11.5	
Gender (male:female)	18:7	81:56	
Subgroups by disease group:			
Total NMD (*n*=128)	19/128 (15%)	109/128 (85%)	
NMD with tracheostomy (*n*=25)	10/25 (40%)	15/25 (60%)	
NMD without tracheostomy (*n*=103)	9/103 (9%)	94/103 (91%)	
NMD with NIV (*n*=18)	1/18 (6%)	17/18 (94%)	
NMD without NIV (*n*=110)	18/110 (16%)	92/110 (84%)	
NMD with gastrostomy (*n*=43)	2/43 (5%)	41 (95%)	
NMD without gastrostomy (*n*=85)	17/85 (20%)	68/85 (80%)	
Total CP GMFCS 4–5 (*n*=34)	6/34 (18%)	28/34 (82%)	^1^*p*= 0.69
CP GMFCS 4–5 with tracheostomy (*n*=8)	3/8 (38%)	5/8 (62%)	
CP GMFCS 4–5 no tracheostomy (*n*=26)	3/26 (12%)	23/26 (88%)	
CP with NIV (*n*=9)	2/9 (22%)	7/9 (78%)	
CP without NIV (*n*=25)	4/25 (16%)	21/25 (84%)	
CP with gastrostomy (*n*=23)	4/23 (17%)	19/23 (83%)	
CP without gastrostomy (*n*=11)	2/11 (18%)	9/11 (82%)	
Subgroups by chronic therapy:			
NIV (*n*=27)	3 (11%)	24 (89%)	^2^*p*= 0.5
Tracheostomy (*n*=33)	13 (39%)	20 (61%)	^3^*p*< 0.05
Gastrostomy (*n*=66)	6 (0.09%)	60 (90.944%)	^4^*p*= 0.06

^1^Chi-square is testing the number of subjects with NMD/number of subjects with CP with PA vs. number of subjects with NMD/number of subjects with CP who did not have PA

^2^Chi-square is testing the number of subjects with tracheostomy/number of subjects without tracheostomy with PA vs. number of subjects with tracheostomy/number of subjects without tracheostomy who did not have PA

^3^Chi-square is testing the number of subjects using NIV/number of subjects not using NIV with PA vs. number of subjects using NIV/number of subjects not using NIV who did not have PA

^4^Chi-square is testing the number of subjects with gastrostomy/number of subjects without gastrostomy with PA vs. number of subjects with gastrostomy/number of subjects without gastrostomy who did not have PA

The age range for the full cohort was 2 months to 17 years (mean 11.7 years) with 99 males and 63 females. Twenty-seven were using non-invasive ventilation (NIV) via a facemask, 10 were ventilated via tracheostomy, 23 had a tracheostomy with no ventilation and 66 had a gastrostomy tube for feeding. Of the 25 patients that had a PA isolate, 19 (76%) had NMD and 6 (24% had CP. Thirteen (52%) had a tracheostomy (of whom 10 were receiving overnight ventilation), 3 (12%) were using NIV and 6 (24%) had a gastrostomy.

There was no significant association between diagnosis of NMD or CP and PA analysis, There was no significant association between use of NIV or gastrostomy and PA analysis but there was a significant association between presence of a tracheostomy and PA positive samples (*p*<0.05).

After exclusion of duplicate samples (from the same patient during the same clinical episode), 62 PA positive clinical episodes were included from the 25 individual patients. Table 2 shows the clinical outcomes of the episodes treated with antibiotics specific to PA, those treated empirically with non-specific antibiotics and those that received no antibiotics for the 60 episodes for which an outcome could be identified. There was no significant association between type of antibiotic and clinical outcome (*p*=0.06).

**Table 2:** Clinical outcomes based on decision to treat (*n*=60). There was no significant association between type of antibiotic and clinical outcome. *p*=0.06 (chi-square)

Outcome	No antibiotics	Non-specific antibiotics	PS specific antibiotics
Did not deteriorate	25 (89%)	10 (59%)	11 (73%)
Deteriorated	3 (11%)	7 (41%)	4 (27%)
Total	28	17	15

There were 20 clinical episodes in 13 individual patients who did not have a tracheostomy. Table 3 shows their clinical outcomes. The children that had a PA isolate without a tracheostomy represented 9/19 of those with NMD (16 clinical episodes) and 3/6 of those with CP (4 clinical episodes).

**Table 3:** Clinical outcomes based on decision to treat (*n*=20) in only those patients without a tracheostomy. There was no significant association between type of antibiotic and clinical outcome. *p*= 0.36 (chi-square)

Outcome	No antibiotics	Non-specific antibiotics	PS specific antibiotics
Did not deteriorate	2 (40%)	7 (64%)	1 (25%)
Deteriorated	3 (60%)	4 (36%)	3 (75%)
Total	5	11	4

Table 4 illustrates the ten episodes in which antibiotics were started or changed after clinical deterioration to one that was specific and sensitive for the PA isolated during the admission after receipt of microbiology results. Thereafter all patients improved.

**Table 4:** Clinical outcomes in PA positive clinical episodes in which patients deteriorated and had antibiotics changed to PA specific antibiotics based on change in treatment after microbiology result (*n*=10)

Outcome	Initial treatment: No antibiotics	Initial treatment: Non-specific antibiotics
Improved after change	2	8
Deteriorated further	0	0
Total	2	8

**Fig. 3:**
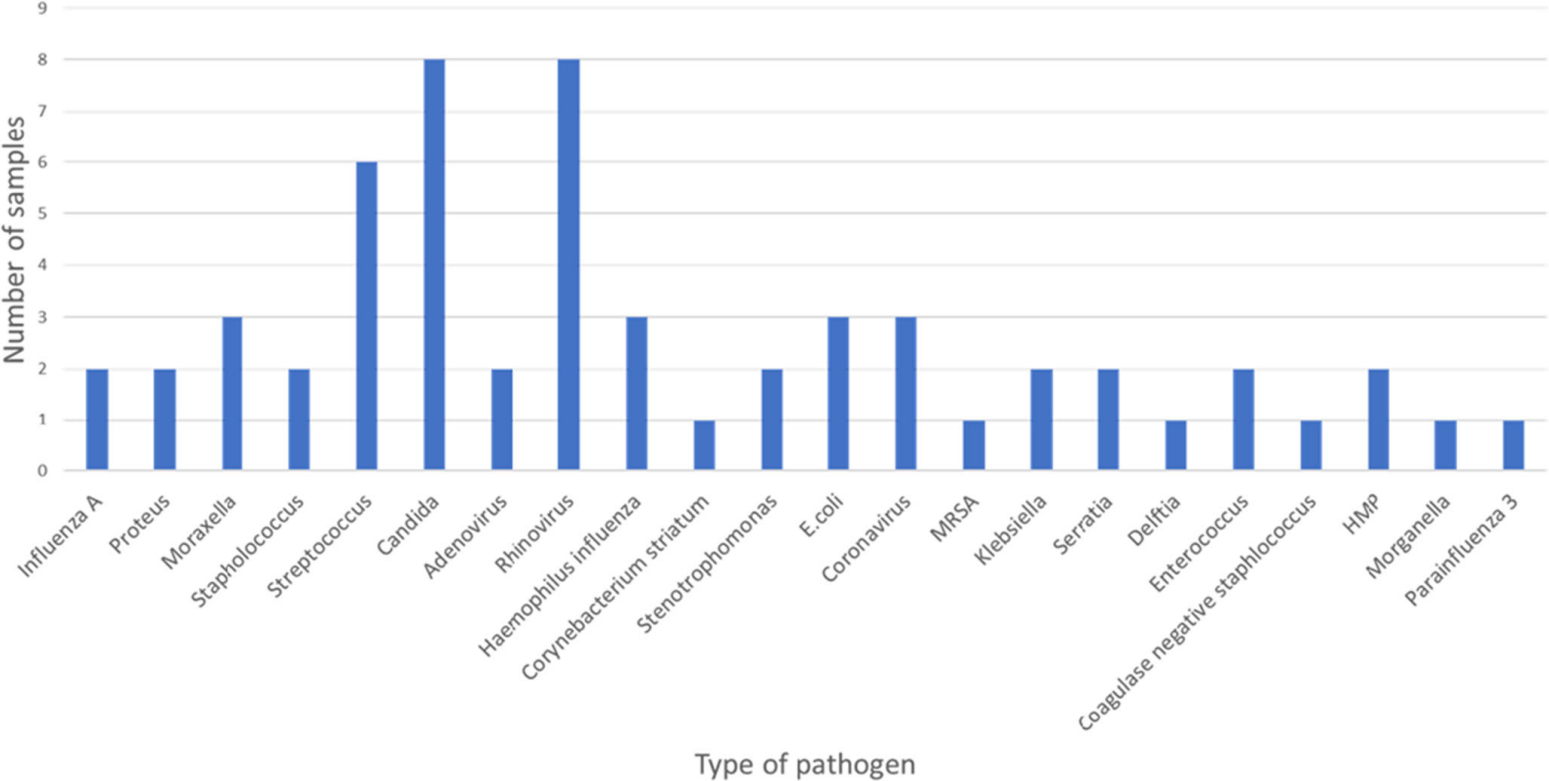
Additional pathogens grown in addition to PA

In 52% samples, multiple pathogens co-existed (Fig. 3).

Tables 5, 6 and 7 show the results of the association between isolated PA infections and multiple pathogens, new PA infection and prior colonisation and the use of prophylactic nebulised colomycin.

**Table 5:** Association between deterioration and presence of PA alone or multiple respiratory pathogens (*p*=0.64)

	PA alone	Multiple pathogens	Total
Clinical deterioration	6	9	15
No clinical deterioration	22	25	47
Total	28	34	62

**Table 6:** Association between deterioration and presence of PA in previous respiratory samples (*p*=0.0002)

	Previous PA	New PA	Total
Clinical deterioration	11	4	15
Clinical deterioration	47	0	47
Total	58	4	62

**Table 7:** Association between deterioration and prior use of nebulised colomycin (*p*=0.19)

	Colomycin	No colomycin	Total
Clinical deterioration	1	14	15
Clinical deterioration	12	35	47
Total	13	49	62

Of the 62 episodes, 28 samples grew PA alone and 34 grew multiple pathogens. In 58 episodes, PA had been grown on previous respiratory samples and of those, nebulised colomycin was being used in 13 episodes. There was no significant association between clinical deterioration and the presence of PA alone or multiple respiratory pathogens. There was a significant association between the presence of PA in previous samples and clinical deterioration with deterioration being more likely to occur in those that had previously grown PA. There was no significant association between clinical deterioration and the use of colomycin nebulisers, although most (12/13) patient taking colomycin did not deteriorate.

In the 15 episodes in which there was a clinical deterioration, there was no association between antibiotic choice and presence of single or multiple pathogens, previous PA growth or use of colomycin nebulisers.

No adverse clinical effects were documented as a direct result of the antibiotics. Of the 62 PA positive clinical episodes, 26 episodes were associated with PA organisms that were documented as being resistant to one of more antibiotics. These episodes were from 8 individual patients, all of whom had recurrent admissions. Table 8 shows the bacteriological and clinical outcomes of these patients.

**Table 8:** Bacteriological and clinical outcomes of patients with antibiotic resistant PA. *HDU*, high dependency unit; *PICU*, paediatric intensive care unit. Antibiotic resistance: resistance pattern during treatment of PA episode described. Bacteriological outcome: resistance patterns of subsequent growths of PA after treatment

	Antibiotic Resistance	Treatment Changes	Bacteriological Outcome	Clinical outcome
Patient 1	Ceftazidime	Changed to tazocin	Resistant to gentamicin	Improvement
Patient 2	Fully sensitive	Treated with cipro	Resistant to gentamicin	Escalated to HDU
Patient 3	Multiresistant	Treated with series of antibiotics, including: tazocin, tobramycin, meropenem	Remained multiresistant	Multiple PICU admissions
Patient 4	Meropenem	Tazocin	Multiresistant	Escalated to PICU
Patient 5	Fully sensitive	Cephalexin	Resistant to cipro	Stayed at home
Patient 6	Multiresistant	Meropenem, tazocin	Multiresistant	Escalated to PICU
Patient 7	Fully sensitive	Treated with series of antibiotics, including: tazocin, ciprofloxacin, meropenem	Multiresistant	Escalated to PICU
Patient 8	Fully sensitive	None	Multiresistant	Stayed at home

## Discussion

This study has shown that of 226 patients with MND or severe CP under the care of a tertiary Children’s Trust, 162 had a respiratory sample sent during a 12-month period and 25 (15%) of these had one or more PA isolates. There was a significant association with presence of a tracheostomy (*p*<0.05). In 52% samples multiple pathogens co-existed. Whilst in 10 clinical episodes, there was an improvement after commencing PA specific antibiotics; of the 60 PA positive clinical episodes, there was no significant association between choice of antibiotic and clinical outcome.

The finding that tracheostomy samples are associated with PA isolates is consistent with other studies that show a predominance of Gram-negative organisms in this patient population [21–25]. Biofilms are formed when the bacteria adhere strongly to the surfaces of the tubes, providing protection to the bacteria against antibiotic treatment. The relationship between presence of PA in the airway and respiratory health or the need for antibiotic treatment is not fully understood. Data from Russel et al. suggest that the early colonisation with *P. aeruginosa* after tracheostomy is associated with higher morbidity [26]; however, McCaleb et al. [27] suggest a poor correlation, reflected by current practice in which many patients are only treated with antibiotics when they are symptomatic and there is clear clinical evidence of bacterial infection.

The majority of patients did not have a significant clinical deterioration regardless of treatment choice and the largest group had no treatment. This may reflect the possibility that some of these samples could have been taken for surveillance purposes, although this is not routine practice in our centre [28]. Alternatively, if patients were only mildly unwell when samples were taken, it is likely that the clinician would have a watch and wait approach, to avoid unnecessary morbidity associated with PA treatment. Whilst information on additional cultures such as urine was not collected, a large number of co-existing pathogens were identified and there was no significant association between clinical deterioration and multiple respiratory pathogens. It is difficult to draw conclusions from this, however, as the microbiological cause of those that did deteriorate remains unclear. It is possible that for many, the relatively mild nature of their illness was predominantly the natural history of a viral infection, indicating that the PA was not the pathogen driving the illness, and merely reflected colonisation. There was a significant association between the presence of PA in previous samples and clinical deterioration with deterioration being more likely to occur in those that had previously grown PA. This perhaps indicates that deterioration in this chronically ill patient group was more likely associated with the pre-existing poor condition of the child.

The largest group to deteriorate was that in whom non-specific antibiotics were initially given, but numbers were small and therefore little conclusion can be drawn from these figures. Due to the small sample size *p* values were not adjusted for multiple comparisons. The improvement seen in the ten patients that had PA-specific antibiotics started following microbiology results may indicate that the ability to isolate a specific pathogen earlier in the illness may prevent further deterioration. It was not possible to ascertain from this study whether the use of PA specific antibiotics early in the illness prior to receipt of microbiology results was detrimental, but no adverse effects were documented as a direct result of the antibiotics. Antibiotic resistant PA organisms were isolated from eight patients, with those developing resistance or remaining multiresistant being more likely to require escalation to intensive care treatment. These findings cannot be attributed to the antibiotic alone, as improvement with time and other treatment changes will have contributed to the outcome.

The retrospective nature of our case note analysis was a limitation of this study which precludes drawing conclusions as to the timing of treatment aimed at PA and prior or new colonisation (as surveillance cultures are not routine practice). More definitive analysis would be enabled by a prospective study to look at outcomes in more detail as well as other possible confounders including the severity of neurodisability and co-morbid conditions. In the absence of clinical guidelines many of the results were reliant on individual clinician judgements in terms of IV versus oral antibiotics and whether broad-spectrum or PA specific antibiotics were commenced, creating a potential for bias. In the patients that did not have a respiratory sample sent for analysis, it was assumed that this was due to good respiratory health and absence of clinical indicators to raise concerns for infection. As none of these clinically well patients were actively sampled, the results of this study may underestimate the true prevalence.

In conclusion, in a 12-month retrospective study of patients with NMD or CP, the majority of the 15% patients with respiratory PA isolates did not significantly deteriorate clinically and outcomes in relation to antibiotic treatment choices were unclear due to small patient numbers. A large number of co-existing pathogens was identified and it is possible that for many the relatively mild nature of their illness was predominantly the natural history of a viral infection, indicating that the PA was not the pathogen driving the illness. The ability to identify the presence of PA early in the illness may have resulted in earlier clinical improvement in the patients that were initially treated with non-specific antibiotics. Antibiotic resistance in patients with recurrent admissions could be reduced by introducing targeted antibiotic treatment, although this study was not able to show this. A larger prospective study may establish clearer criteria for guideline development and more sophisticated methods to direct treatment choices are needed. Techniques such as the ability to identify virulent strains of PA using point-of-care testing may improve patient outcomes and prevent the development of antibiotic resistance in the future.

## Data Availability

Available on request

## Abbreviations

CP: Cerebral palsy
GMFCS: Gross Motor Function Classification System
HDU: High dependency unit
IV: Intravenous
LTV: Long-term ventilation
NIV: Non-invasive ventilation
NMD: Neuromuscular disease
PA: *Pseudomonas aeruginosa*
PICU: Paediatric intensive care unit
